# Investigating perceptual discrimination thresholds for attributes of whole-body vibration

**DOI:** 10.1038/s41598-026-40033-4

**Published:** 2026-02-17

**Authors:** Berkay Kullukcu, Jonas Krautwurm, Sebastian Merchel, Robert Rosenkranz, Ercan Altinsoy

**Affiliations:** 1 Institute of Acoustics and Speech Communication, TU Dresden Chair of Acoustics and Haptics, Dresden, 01069 Saxony Germany; 2 Centre for Tactile Internet with Human-in-the-Loop (CeTI), TU Dresden, Dresden, 01069 Saxony Germany; 3 Research Cluster 6G-life, TU Dresden, Dresden, 01069 Saxony Germany

**Keywords:** Haptic perception, Vibrotactile feedback, User-centered design, Engineering, Neuroscience

## Abstract

Understanding the limitations of haptic perception in humans is critical for the successful design of effective haptic feedback systems, however, it is unclear how perceived discrimination thresholds relate to specific qualitative perceptual attributes. In this work, it was aimed to estimate quantitative perceptual discrimination thresholds for six qualitative attributes of whole-body vibration, which included “weak”, “up-and-down”, “tingling”, “repetitive”, “even”, and “fading”. A series of experiments were conducted using a multimodal vibration platform and an electrodynamic shaker, where participants performed a magnitude estimation task that systematically varied physical parameters (intensity, frequency, modulation, decay rate) from reference measures to assess perceived discrimination thresholds, testing each attribute separately. Unique and attribute-specific relationships between physical parameters and perceptual insights were found. Key findings include a consistent just-noticable difference in level threshold ($JNDL_{\pm }$) of $\le$ 2 dB for the “weak” attribute, a lower frequency threshold ($JNDF_-$) between 10 - 20 Hz for “tingling” (at a 120 Hz reference) and $\le$ 5 Hz (at a 30 Hz reference) for “up-and-down”, a lower modulation frequency threshold ($JNDF_-$) between 0.2 - 0.4 Hz for “repetitive” (at a 2.4 Hz reference), a lower bandwidth threshold ($JNDF_-$) between 1 - 2 Hz for “even” (at a 3 Hz reference), and a threshold ($JND_+$) of $\le$ 0.5 for the decay rate $\delta$ (at a 0.5 reference) for “fading”. The findings represent quantitative evidence to establish a framework for relating specific physical signal parameters to qualitative perceptual attributes and also inform the design of perceptually aligned haptic systems that match haptic feedback to human sensory limits.

Vibrotactile stimuli are important in human-computer interaction, virtual reality, and product design by intuitively providing feedback through tactile sensations. This type of feedback can be found now everywhere, from the game controllers and VR controllers that enhance immersion to the mobile phones and advanced taptic engines in smart devices that provide notifications and haptic cues. To be able to develop a better user experience, one needs to understand how users perceive changes in vibration, which are often quantified as the Just-Noticeable Difference (JND)^1^. Furthermore, effective vibrotactile feedback is based on how users perceive and describe vibrations in terms of everyday, relevant experiences^2,3^. This becomes very critical in areas like automotive systems and virtual environments, where user experience and immersion are crucial.

Many forms of vibration encountered in everyday life, such as whole-body vibrations (WBV), carry crucial sensory information. For instance, WBV is used in applications to present road or vehicle dynamics by providing feedback through the seat of a vehicle^4^. While the use of sinusoidal signals for spatial cueing has been demonstrated in driving assistance systems, more general approaches utilizing temporal and spectral properties are still under investigation^5^. The temporal and spectral characteristics of vibration are the basis for the description of perceptual attributes that are understandable to non-professionals^6^.

The role of vibration perception is particularly significant in product design, where user expectations and perceived quality play a major role. Predictive models linking vibration characteristics to attributes such as comfort, quality, and sportiness could transform design processes^7^. These insights are crucial for virtual reality applications, where vibration feedback contributes to the plausibility illusion^8^ , the feeling that the virtual environment is real, enabling users to interact naturally with virtual environments. Achieving this illusion requires careful alignment of tactile feedback with user expectations, especially in more complex virtual environments^8,9^.

It is also important to establish the broader importance of the JND as the core metric for this work^10–12^. The study aims to build a JND framework for qualitative vibration attributes. This approach is based on the widely usage of JND-based frameworks in other sensory domains, which have been important in optimizing user perception and system efficiency. The JND is an important metric that covers the measurement of perceptual thresholds beyond vibrotactile perceptions, as it informs the design and calibration of a wide variety of other sensory displays. In developing electrotactile displays, for example, the JND of intensity, frequency, and duration are all referred to in calibrating usability for sensory feedback systems^13^. Research in this area has provided a critical distinction; when stimuli are temporally presented with some spacing, JND values are much lower, indicating better perceptual sensitivity, and discrimination is worse, involving larger JNDs, when the parameters are modulated continuously^14^. This distinction is important for calibrating closed-loop applications, because traditional JND values can overestimate perceptual sensitivity in the dynamic applications of real-world environments and dynamic systems^13,14^. JND characteristics are also fundamental to optimizing visual displays. In medical imaging research, JND standards offer extended grayscale requirements for ultra-low luminance displays, which can help to provide a more accurate diagnostic capability^15^, while high luminance displays can increase the total number of JNDs, thereby improving perceptual visibility under bright ambient conditions^16^. In the consumer device space, algorithms that apply JND information are increasingly used to optimize image luminance to reduce power consumption while maintaining imperceptibility to changes in visual quality^17^.

Despite advancements, a systematic framework for designing and evaluating vibrations to convey specific perceptual attributes is still lacking^18^. Current studies focus on isolated parameters like JND for frequency (JNDF) and JND for level (JNDL), but they fail to connect these thresholds to the perceptual attributes they are associated with^19^. It is within these parameters that most studies on the perception of vibrotactile attributes of intensity, frequency, and modulation, has been conducted. Works, for example^20^, which identified important perceptual attributes in creating realistic virtual-environment tactors, including “tingling” and “fading,” do not report on small, detectable differences among human subjects. Also, the research conducted in^21^ studied frequency discrimination in low-frequency ranges but did not investigate how various attributes interact with other parameters like decay or bandwidth. This study attempts to fill these gaps by linking JNDF and JNDL thresholds, which are rooted in physical parameters, to their perceptual counterparts, thereby enabling a unified framework for designing intuitive tactile feedback.

While traditional signal-based descriptors, such as root mean square and center frequency, have their own merits, they usually don’t capture the complexity of natural vibrations and match non-expert subjective perceptions. Recent works have explored the perceptual dimensions across various frequency bands; however, there is still a lack of systematic investigation that connects those dimensions to tactile perception^22^. For example, tactile perception is influenced not only by mechanical properties but also by subjective user expectations and contextual factors^23^.

The current study extends directly from the work of Rosenkranz & Altinsoy^20^ that established the perceptual-attribute framework for vibration. The work is a sensory-perceptual representation of the vibration sensation, but it doesn‘t report on the extent of human sensitivity to changes to vary the specific qualitative attributes. This study addresses that gap. The goal is to continue the inclusion of the framework by attempting to report on the perceptual discrimination thresholds (identified as JND ranges) for this experiment to obtain the proper representation of perceptually based haptic design.

The study is guided by two primary research questions. First, (RQ1) What are the perceptual discrimination thresholds (JNDs) for the qualitative attributes “weak,” “up-and-down,” “tingling,” “repetitive,” “even,” and “fading”? Second, (RQ2) How are the distinct perceptual thresholds related to their respective underlying physical parameters (amplitude, frequency, modulation frequency, bandwidth, and decay rate)? To answer these questions, it is necessary first to define the specific perceptual attributes under investigation. The following section details the selection and justification of the six key attributes chosen for this study, and provides the foundation for the experimental design and methodology.

## Selecting the sensory attributes for vibration discrimination

The selection of perceptual attributes is crucial for developing tactile systems that aligns with the vibration discrimination capabilities and enable intuitive, user-centered interactions. These attributes connect the physical parameters of vibrotactile stimuli, and the subjective experiences they create, to build haptic feedback that aligns with human perception in everyday contexts. By connecting these attributes with the physical parameters, the approach supports user experiences across various platforms, from handheld devices to full-body interfaces^20,24^.

Six key attributes: “weak”, “up-and-down”, “tingling”, “repetitive”, “even”, and “fading”, were chosen based on their prevalence in the literature^20^. The attributes are linked to specific spectral/temporal features, enabling the translation of user expectations into physical vibration parameters. For example, “tingling” and “fading” increases immersion in virtual environments by providing subtle, plausible feedback^24,25^, while “repetitive” describes periodic modulation that help people distinguish time-invariant from transient tactile stimuli.

These attributes also demonstrate cross-domain applicability, ranging from automotive systems (e.g., road condition feedback) to gaming controllers (e.g., dynamic interactions). “up-and-down” is suitable for dynamic scenarios, while “even” is responsible for creating a consistent and predictable feedback. These attributes‘ intuitive characteristics make haptic systems more accessible, particularly in assistive devices, where tactile feedback serves as a communication channel for humans^6,24^.

Each stimulus type was chosen to isolate the specific physical parameter under investigation, based on their psychophysical connections. The attributes “weak”, “up-and-down”, and “tingling” are fundamental perceptual responses to intensity and frequency, respectively. Therefore, a sinusoidal signal was used, as it is the simplest waveform, allowing itself to test these attributes by varying only amplitude (for “weak”) or a single frequency (for “up-and-down” and “tingling”) without making the variable changes too much complex. In contrast, “repetitive” describes a periodic, rhythmic sensation, which is most directly generated and controlled by the modulation frequency of an amplitude-modulated (AM) signal. “even” relates to the perceptual smoothness or fullness of a vibration rather than a pure tone. This is a function of spectral content, making narrowband noise the ideal stimulus, as its bandwidth can be precisely controlled. Finally, “fading” is a purely temporal attribute, so an impulse stimulus was used, as its decay rate ($\delta$) is the explicit physical parameter that controls the decay characteristic.

## Finding the perceptual discrimination thresholds for the sensory attributes

### Experimental design

The experiment employed a magnitude estimation task to estimate the perceptual discrimination thresholds (defined as JND ranges) for each perceptual attribute. This approach was chosen as it is a classic psychophysical method for directly measuring subjective sensory experiences, as founded by Stevens and discussed in psychophysical literature^26^. Participants were seated in a Recaro racing seat and were presented with WBV stimuli through the hydraulic platform and an electrodynamic shaker.

For each attribute, participants first experienced a reference vibration, which was assigned a rating value of 100 points for the tested attribute. They were then presented with test vibrations that had incremental changes in the relevant physical parameters (e.g., frequency, amplitude, decay rate). Participants judged the test vibrations relative to the reference and rated their perceived differences.

The experiment was conducted over six sessions, each lasting approximately eight to ten minutes, to maintain participants’ attention and ensure reliable data collection. Across all sessions, each participant evaluated a total of 66 distinct test stimuli (as detailed in Table 1), with each stimulus being compared to its corresponding reference. This resulted in a total number of trials of: (11 Weak + 12 Up-and-Down + 10 Tingling + 11 Repetitive + 12 Even + 10 Fading) x 2 repetitions x 11 participants = 1342 trials^27^. All stimuli were presented in a randomized order, and participants could repeat the stimuli as needed. The study was approved by the Ethics Committee of the Technische Universität Dresden (SR-EK-111032020-Amendment) and conducted in line with guidelines of the Helsinki Declaration.

### Stimuli

A set of WBV stimuli was designed to investigate six perceptual attributes. The stimuli were generated using a multimodal vibration platform and an electrodynamic shaker, covering frequencies ranging from 1 Hz to 300 Hz. Table 1 summarizes the physical parameters and ranges for each attribute. Sinusoidal, amplitude-modulated, narrowband noise, and impulse vibrations were used to represent these attributes. These reference values were selected based on two primary criteria. First, they were identified as clearly perceptible and representative examples of the qualitative attribute in our previous foundational work^24^, and secondly, they were chosen to be sufficiently far from the edges of the platform’s operating range to allow for testing comparison stimuli both above and below the reference point.

**Table 1:** Summary of stimulus parameters for perceptual attributes.

Attribute	Stimulus Type	Frequency Range (Hz)	Amplitude Range (dB SL)	Additional Parameters	Reference Stimuli	Test Stimuli
Weak	Sinusoidal	50	10–30	-	26 dB	30, 28, 24, 22 dB
					18 dB	20, 16 dB
					14 dB	12, 10 dB
Up-and-Down	Sinusoidal	12–100	20	-	17 Hz	12, 20, 22 Hz
					30 Hz	25, 35 Hz
					55 Hz	45, 65, 75, 100 Hz
Tingling	Sinusoidal	100–300	25	-	120 Hz	100, 110, 130, 140 Hz
					225 Hz	200, 250, 275, 300 Hz
Repetitive	Amp. Modulated	50 (carrier)	25	Modulation Freq.: 2–10 Hz	2.4 Hz	2, 2.2, 2.8 Hz
					3.6 Hz	2.8, 3.9 Hz
					5 Hz	2.5, 7.5, 10 Hz
Even	Narrowband Noise	50 (center)	25	Bandwidth: 0–25 Hz	3 Hz	0, 1, 2, 4, 5, 6 Hz
					10 Hz	8, 15, 20, 25 Hz
Fading	Impulse	5	Varying	Decay Rate ($\delta$): 0.5–4.5	$\delta = 0.5$	$\delta = 1$
					$\delta = 2$	$\delta = 1.5, 2.5$
					$\delta = 4$	$\delta = 2.5, 3, 3.5, 4.5$

### Experimental setup

**Fig. 1:**
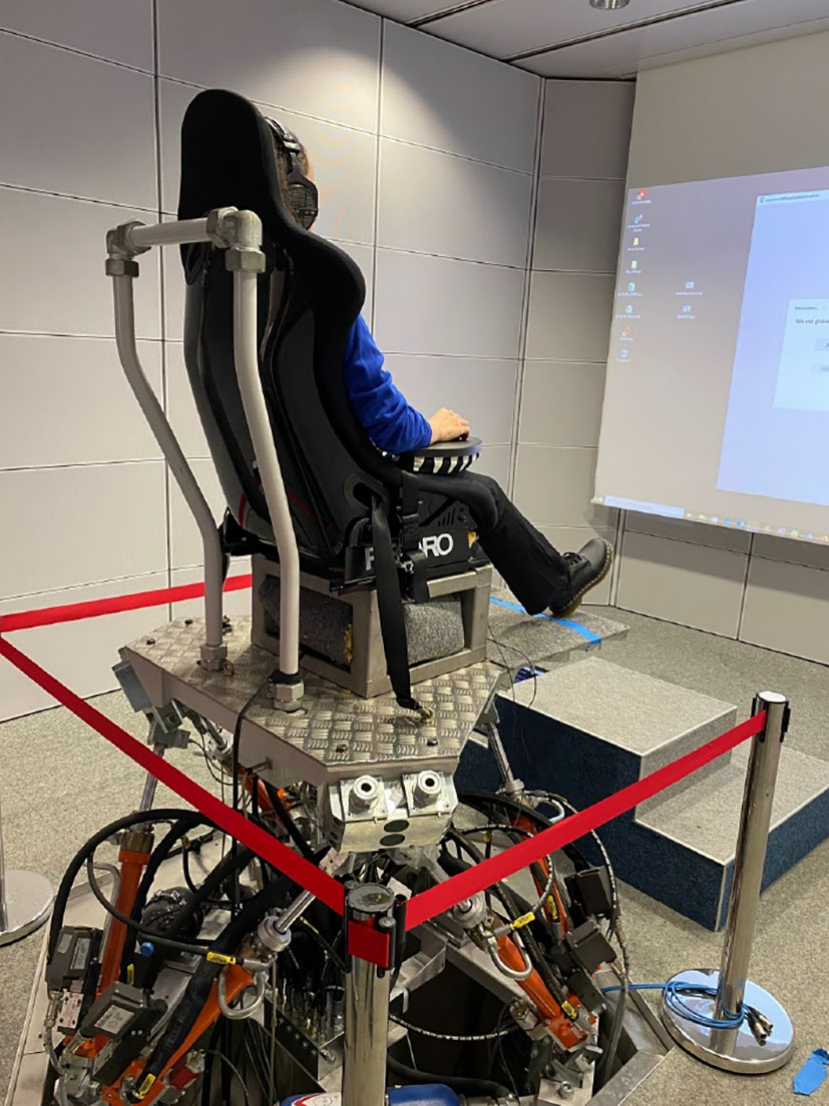
Vibration platform in the Multimodal Measurement Laboratory (MMM).

The vibrational capabilities of the Multimodal Measurement Laboratory (MMM) at TU Dresden were used in the experiment to investigate JNDF and JNDL thresholds. The laboratory features a six-degree-of-freedom (DOF) hydraulic motion platform and an electrodynamic shaker system, as shown in Fig. (1), allowing precise control of both low- and high-frequency vibrations^28^. The hydraulic motion platform generates low-frequency vibrations ranging from 1 to 9 Hz, for studying whole-body tactile interactions. To minimize interference, the system operates on a separate ferro-concrete foundation and uses hydraulic components such as low-noise axial pumps and dynamic valves. The platform is capable of producing both gradual and sudden variations in vibration intensity, which can deliver low-frequency tactile stimuli during the experiment.

To extend the frequency range, an electrodynamic shaker was used to generate high-frequency vibrations above 9 Hz. The separation of frequencies between the hydraulic system and the electrodynamic shaker was achieved using a uniquely designed software toolbox. This system enables the generation of localized vibrations, allowing for the research of WBV perceptual attributes also at higher frequencies. Together, these systems cover a broad spectrum of tactile frequencies. To ensure the generalization of the results, calibration of the reproduction system under experimental conditions was necessary^29^ to account for individual differences in participants’ physical characteristics, such as weight and body composition, which can affect vibration perception. For this purpose, individual body transfer functions were measured for all participants using a finite impulse response (FIR) filter of the inverse transfer function, as described in^30^.

### Participants

A total of 11 participants (9 male, 2 female) aged 21–45 years took part in the experiment. All participants had no known tactile impairments and prior experience in vibrotactile experiments. The study was conducted with the understanding and written consent of each participant. All stimuli of the study showed vibration levels below the one hour exposure threshold defined in^31^ which was the approximate experiment duration.

### Results

**Fig. 2:**
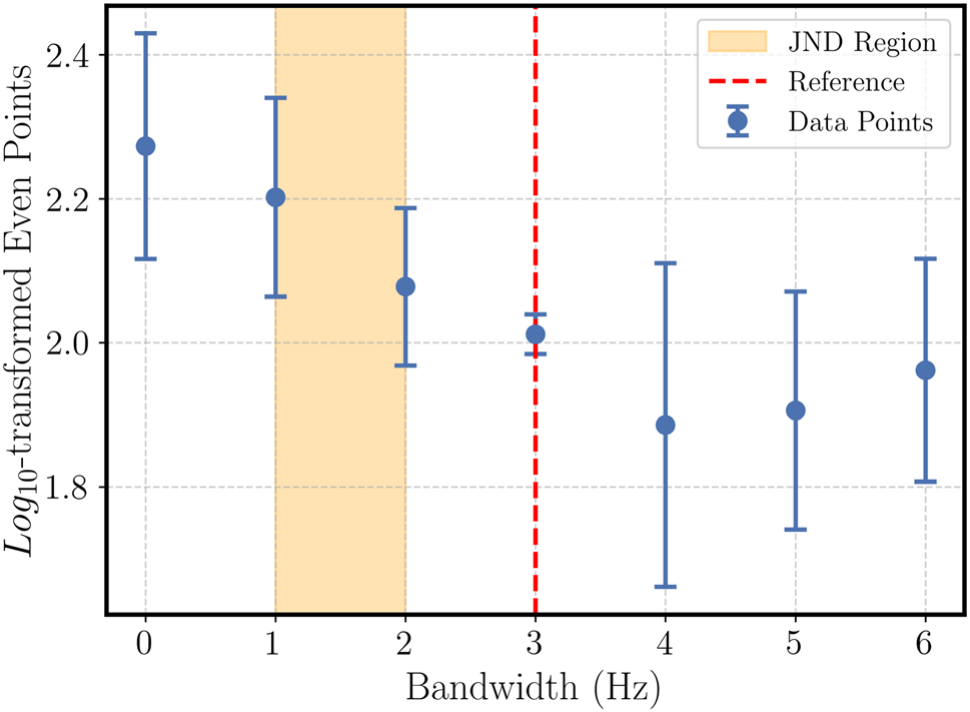
Mean $log_{10}$-transformed magnitude estimation ratings for the “even” attribute as a function of bandwidth. The shaded area represents the estimated JND range ($p > 0.05$ compared to the 3 Hz reference).

**Fig. 3:**
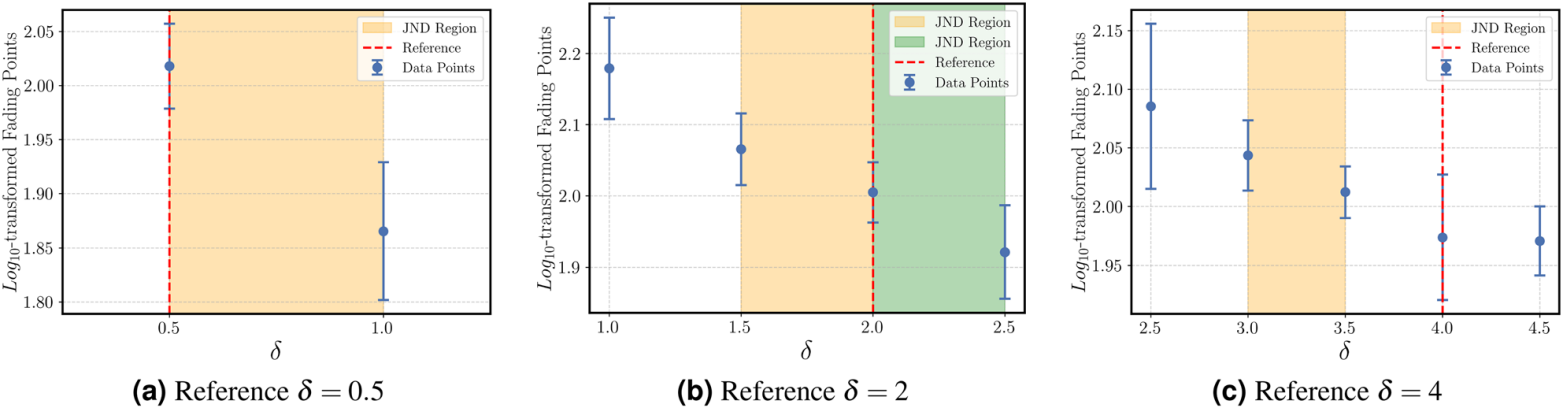
Mean $log_{10}$-transformed magnitude estimation ratings for the “fading” attribute as a function of decay rate ($\delta$). Shaded areas represent the estimated JND ranges for each reference.

**Fig. 4:**
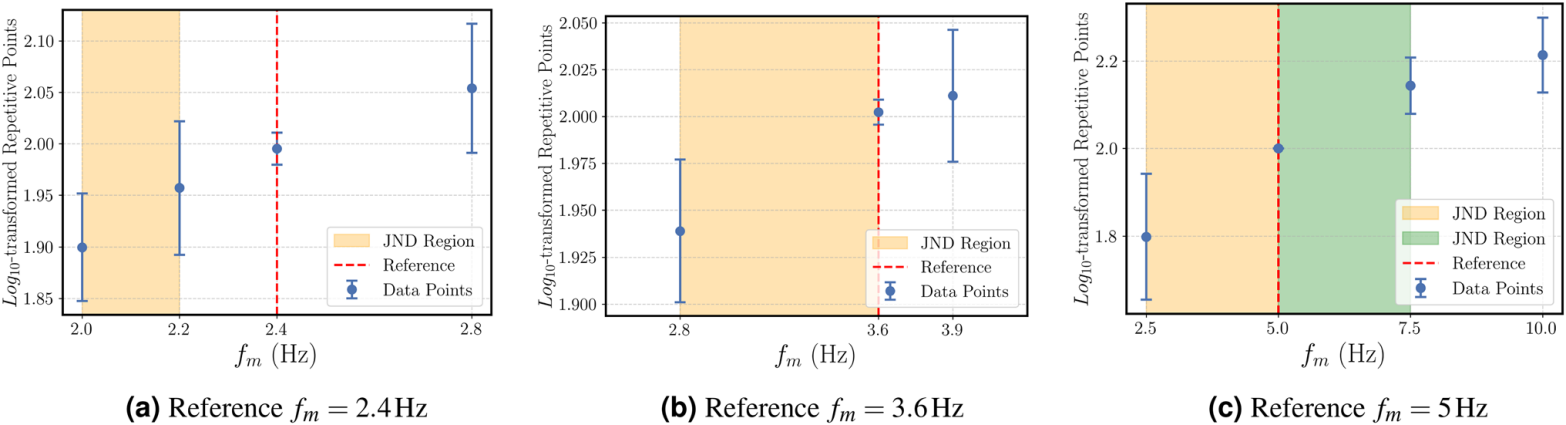
Mean $log_{10}$-transformed magnitude estimation ratings for the “repetitive” attribute as a function of modulation frequency ($f_m$). Shaded areas represent the estimated JND ranges for each reference.

**Fig. 5:**
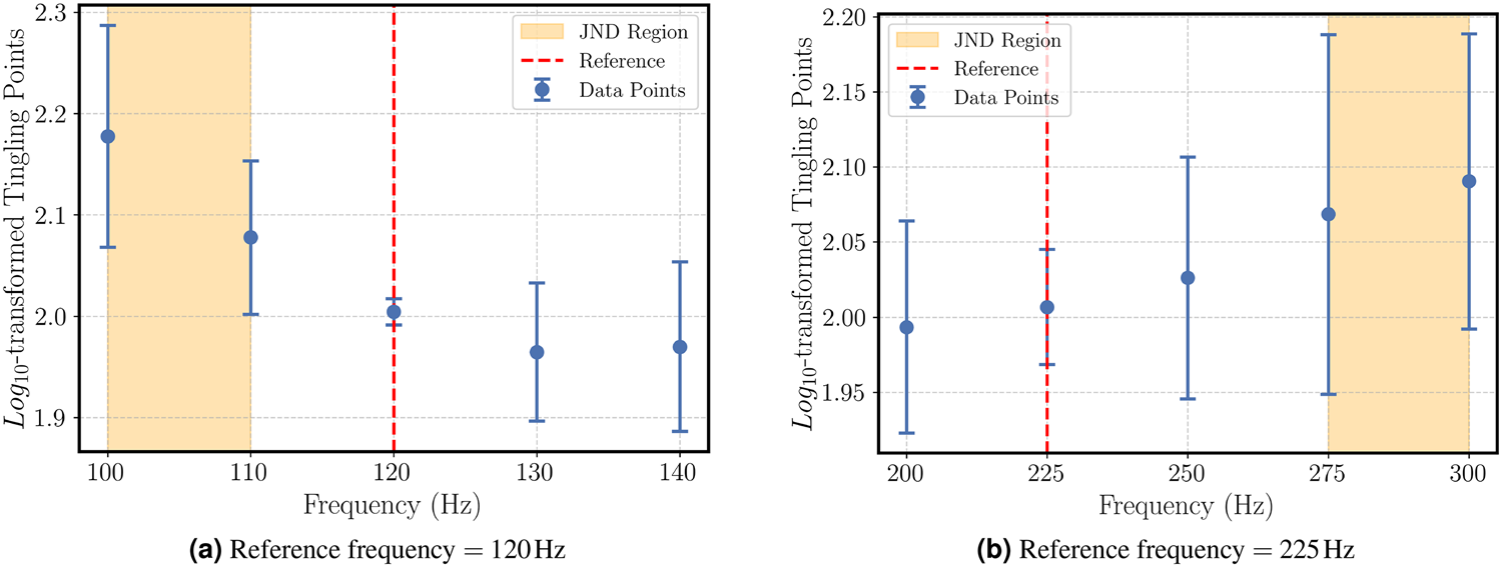
Mean $log_{10}$-transformed magnitude estimation ratings for the “tingling” attribute as a function of frequency. Shaded areas represent the estimated JND ranges for each reference.

**Fig. 6:**
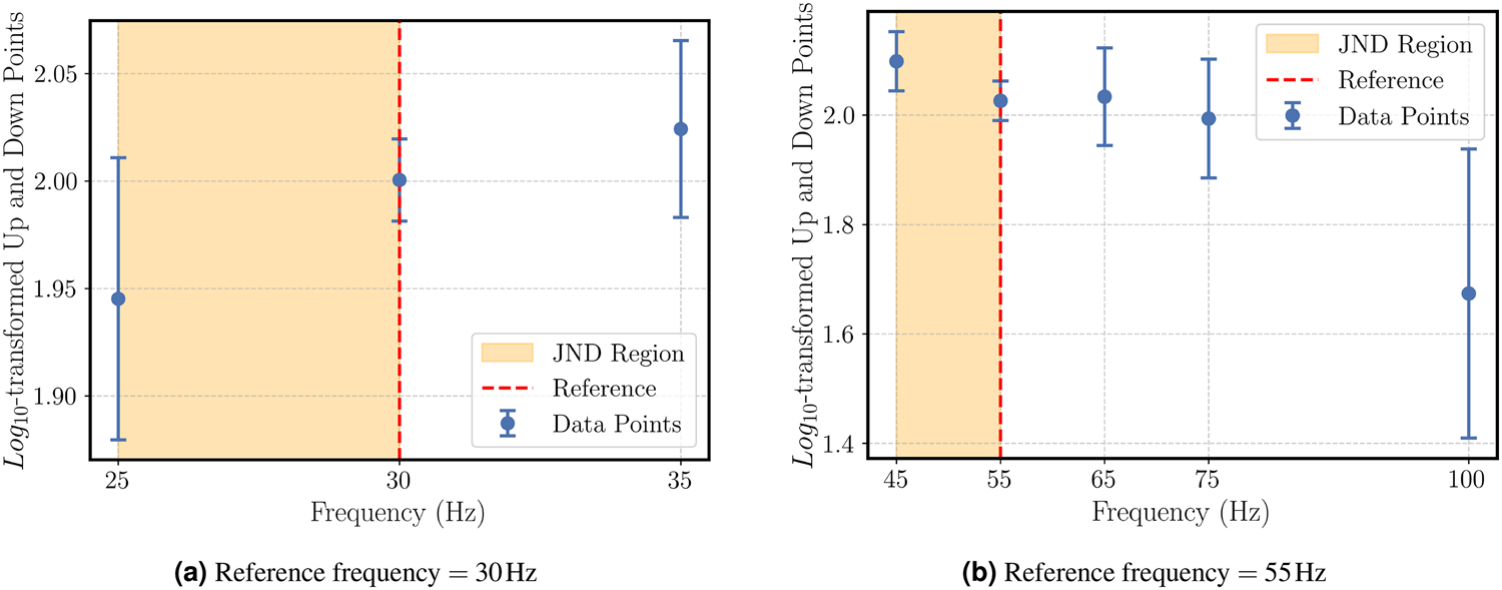
Mean $log_{10}$-transformed magnitude estimation ratings for the “up-and-down” attribute as a function of frequency. Shaded areas represent the estimated JND ranges for each reference.

**Fig. 7:**
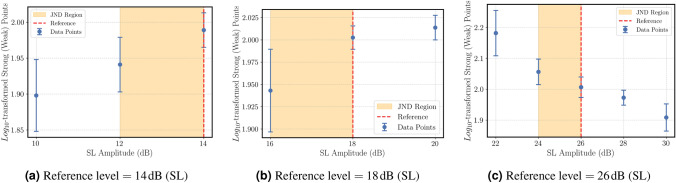
Mean $log_{10}$-transformed magnitude estimation ratings for the “weak” attribute as a function of amplitude level. Shaded areas represent the estimated JND ranges for each reference.

The results of the magnitude estimation experiments give information about the estimated JND ranges for six perceptual attributes, which show how participants perceived changes for different vibration parameters. To identify the perceptual threshold ranges, the Wilcoxon signed-rank test, a standard non-parametric method for analyzing paired, non-normally distributed data^32^, was used to analyze perceptual paired differences among participant answers. For each attribute, the null hypothesis, stating no perceptual difference compared to the reference, was tested against the alternative hypothesis that participants could detect a change. This approach enabled the detection of areas where noticeable differences in the attributes were perceived, allowing for an estimation of the threshold ranges. To interpret the results, the JND range (visualized in Figures 2-7) was defined as the region of stimulus values around the reference for which the null hypothesis could not be rejected ($p > 0.05$). Consequently, the reported JNDF and JNDL metrics represent the upper limits of these perceptual threshold ranges, identified as the first comparison stimulus outside the range that showed a statistically significant perceptual difference from the reference ($p < 0.05$). Where applicable, thresholds are denoted as $JND_+$ (or $JNDF_+/JNDL_+$) for a perceptible increase above the reference, and $JND_-$ (or $JNDF_-/JNDL_-$) for a perceptible decrease below the reference. The magnitude estimation results were normalized using a $log_{10}$ transformation. This transformation was applied to account for the nonlinear nature of human perception, in accordance with Weber’s and Fechner’s laws^33,34^.

#### Even

The results are given in Fig. (2) for the reference point with 3 Hz bandwidth of the “even” attribute.

The results are as following:

- For a narrowband noise signal with a reference bandwidth of $BW_{ref}$ = 3 Hz, a $JNDF_-$ of 1 Hz < $\Delta BW \le$2 Hz (p < 0.05) and,
- For a narrowband noise signal with a reference bandwidth of $BW_{ref}$ = 10 Hz, no statistically significant (p > 0.05) JNDF value in close proximity was detected.

#### Fading

The “fading” characteristic of vibrational signals was modified by adjusting the $\delta$ coefficient, which is linked to the damping factor $D$ and the frequency of oscillation $f$. The decay of the signal is changed by an exponentially decaying term, influenced by both the damping factor and the frequency, which together influence at which amplitude the oscillation will decrease over time.

The signal’s amplitude modulation is expressed in the following Eq. (1):

1 $$\begin{aligned} a(t) = \sum _{n} A_n \cdot e^{-D_n \cdot 2 \pi f t} \cdot \sin (2 \pi f t) \end{aligned}$$

Where:

- $a(t)$ is the amplitude of the signal at time $t$,
- $A_n$ is the amplitude scaling for each oscillating mode,
- $D_n$ is the damping factor for each mode,
- $f$ is the frequency of the signal.

The starting amplitude $A$ is related to the maximum amplitude $a_{\text {Max}}$ by Eq. (2):

2 $$\begin{aligned} A = \frac{a_{\text {Max}}}{e^{(-D \cdot \text {atan}(1/D))} \cdot \sin (\text {atan}(1/D))} \end{aligned}$$

In this equation, the parameter $D$ modulates the initial amplitude and the rate of decay.

The parameter $\delta$, defined in Eq. (3) as:

3 $$\begin{aligned} \delta = 2 \pi f \cdot D \end{aligned}$$

represents the combined influence of frequency and damping on the decay rate. By adjusting $\delta$, the decay behavior of the vibrational signal was modified, with higher values of $\delta$ leading to faster decay. The increase of this parameter creates a reduction in the signal’s energy over time and the oscillations fade more quickly. For each resonating frequency $f$, the decay rate is exponentially governed by the term $e^{-2 \pi f D t}$, thus, the parameter $\delta$ indirectly adjusts the damping factor $D$, and in turn, the decay rate of the signal.

By tuning $\delta$, the decay profile of the vibrational signal can be controlled to better match specific experimental or perceptual requirements. The relationship between $\delta$ and the damping factor $D$, as described above, enables manipulation of the signal’s “fading” characteristics.

The results are given in Figs. (3a)-(3c) for 3 different reference points of the “fading” attribute.

The results are as following:

- For an impulse vibration with a reference decay rate of $\delta _{ref}=0.5$, a $JND_+$ of $\Delta \delta \le 0.5$ $(p<0.05)$ and,
- For an impulse vibration with a reference decay rate of $\delta _{ref}=2$, a $JND_-$ of $\Delta \delta \le 0.5$ $(p<0.05)$ and,
- For an impulse vibration with a reference decay rate of $\delta _{ref}=4$, a $JND_-$ of $\Delta \delta \le 1$ $(p<0.05)$ were detected.

#### Repetitive

The results are given in Figs. (4a)-(4c) for 3 different reference points of the “repetitive” attribute.

The results are as following:

- For an amplitude modulated vibration with a reference modulation frequency of $f_{m,ref}$=2.4 Hz, a $JNDF_-$ of 0.2 Hz < $\Delta f_m \le$0.4 Hz $(p<0.05)$ and,
- For an amplitude modulated vibration with a reference modulation frequency of $f_{m,ref}$=3.6 Hz, a $JNDF_-$ of $\Delta f_m \le$0.8 Hz $(p<0.05)$ and,
- For an amplitude modulated vibration with a reference modulation frequency of $f_{m,ref}$=5 Hz, a $JNDF_{\pm }$ of $\Delta f_m \le$2.5 Hz $(p<0.05)$ were detected.

#### Tingling

The results are given in Figs. (5a)-(5b) for 2 different reference points of the “tingling” attribute.

The results are as following:

- For a sinusoidal signal with a reference frequency of $f_{ref}$=120 Hz, a $JNDF_-$ of 10 Hz < $\Delta f \le$20 Hz $(p<0.05)$ and,
- For a sinusoidal signal with a reference frequency of $f_{ref}$=225 Hz, a $JNDF_+$ of 50 Hz < $\Delta f \le$75 Hz $(p<0.05)$ were detected.

#### Up and down

The results are given in Figs. (6a)-(6b) for 2 different reference points of the up and down attribute.

The results are as following:

- For a sinusoidal signal with a reference frequency of $f_{ref}$=17 Hz, no statistically significant (p > 0.05) JND value in close proximity and,
- For a sinusoidal signal with a reference frequency of $f_{ref}$=30 Hz, a $JNDF_-$ of $\Delta f \le$5 Hz $(p<0.05)$ and,
- For a sinusoidal signal with a reference frequency of $f_{ref}$=55 Hz, a $JNDF_+$ of $\Delta f \le$10 Hz $(p<0.05)$ was detected.

#### Weak

The results are given in Figs.(7a)-(7c) for 3 different reference points of the “weak” attribute.

The results are as following:

- For a sinusoidal signal with a reference level of $L_{ref}=26 dB$ (SL), a $JNDL_+$ of $\Delta L \le 2 dB$ $(p<0.05)$ and,
- For a sinusoidal signal with a reference level of $L_{ref}=18 dB$ (SL), a $JNDL_{\pm }$ of $\Delta L \le 2 dB$ $(p<0.05)$ and,
- For a sinusoidal signal with a reference level of $L_{ref}=14 dB$ (SL), a $JNDL_-$ of $\Delta L \le 2 dB$ $(p<0.05)$ were detected.

Additional statistical measures for the experiment results are given in Table (2).

**Table 2:** Statistical summary of perceptual discrimination thresholds (Estimated JND Ranges) for qualitative attributes.

Attribute	Reference Stimuli	Test Stimuli	Thresholds	P-value	Effect Size*	Wilcoxon W
Even	$BW_{ref}$ = 3 Hz	BW = 1 Hz	$JNDF_-: 1 \text { Hz} < \Delta BW \le 2 \text { Hz}$	0.0039	−0.815	0.0000
Fading	$\delta _{ref} = 0.5$	$\delta = 1$	$JND_+: \Delta \delta \le 0.5$	0.0020	0.960	0.0000
	$\delta _{ref} = 2$	$\delta = 1.5$	$JND_-: \Delta \delta \le 0.5$	0.0156	−0.617	0.0000
	$\delta _{ref} = 2$	$\delta = 2.5$	$JND_+: \Delta \delta \le 0.5$	0.0078	0.741	1.0000
	$\delta _{ref} = 4$	$\delta = 3$	$JND_-: 0.5 \le \Delta \delta \le 1$	0.0078	−0.778	0.0000
Repetitive	$f_{m,ref} = 2.4$ Hz	$f_{m} = 2$	$JNDF_-: 0.2 \text { Hz} < \Delta f_m\le 0.4 \text { Hz}$	0.0039	0.951	0.0000
	$f_{m,ref} = 3.6$ Hz	$f_{m} = 2.8$	$JNDF_-: \Delta f_m\le 0.8 \text { Hz}$	0.0078	0.901	0.0000
	$f_{m,ref} = 5$ Hz	$f_{m} = 2.5$	$JNDF_-: \Delta f_m\le 2.5 \text { Hz}$	0.0078	0.778	1.0000
	$f_{m,ref} = 5$ Hz	$f_{m} = 7.5$	$JNDF_+: \Delta f_m\le 2.5 \text { Hz}$	0.0039	−1.000	0.0000
Tingling	$f_{ref} = 120$ Hz	f = 100 Hz	$JNDF_-: 10 \text { Hz} < \Delta f\le 20 \text { Hz}$	0.0117	−0.778	2.0000
	$f_{ref} = 225$ Hz	f = 300 Hz	$JNDF_+: 50 \text { Hz} < \Delta f\le 75 \text { Hz}$	0.0195	−0.550	5.0000
Up-and-Down	$f_{ref} = 30$ Hz	f = 25 Hz	$JNDF_-: \Delta f\le 5 \text { Hz}$	0.0391	0.605	3.0000
	$f_{ref} = 55$ Hz	f = 100 Hz	$JNDF_+: \Delta f\le 45 \text { Hz}$	0.0078	1.000	0.0000
Weak	$L_{ref}$ = 115 dB	L = 117 dB	$JNDL_+: \Delta L \le 2 \text { dB}$	0.0312	−0.672	0.0000
	$L_{ref}$ = 107 dB	L = 105 dB	$JNDL_-: \Delta L \le 2 \text { dB}$	0.0078	0.815	1.0000
	$L_{ref}$ = 103 dB	L = 101 dB	$JNDL_-: \Delta L \le 2 \text { dB}$	0.0156	0.716	1.0000

*Cliff‘s Delta.

## Discussion

The results of the experiments (Section Results) provide answers to the research questions. Regarding RQ1, the perceptual discrimination thresholds for each attribute were identified. Regarding RQ2, this section demonstrates how these thresholds link directly to their underlying physical parameters. To situate these findings within the broader field, our results can be compared with established psychophysical limits, such as those surveyed by Merchel and Altinsoy^19^. Our finding for the “weak” attribute, a symmetric $JNDL_{\pm } \le 2$ dB, is highly consistent with recognized limits of tactile intensity discrimination. Merchel and Altinsoy^19^ note that tactile JNDLs for whole-body vibration are often found to be between 0.5 dB and 1.75 dB and, like auditory thresholds, tend to decrease as sensation level increases. This consistency validates our methodology and confirms that the perception of ’weakness’ is directly and predictably tied to the classical limits of intensity discrimination. Also, work done in^7^ confirms insensibility to weak tactile cues.

Additionally, the vibrational $JNDF_- \le 2$ Hz for “even” not only corresponds to detectability bandwidth, but it corresponds to a form in which subjects perceive deviation as “uneven” and “unstable,” aligning with observations in^35^ about spectral consistency. In a similar manner, for the decay rate based on the findings of “fading”, a threshold of $\le 0.5$ corresponds with subjects’ report of “dissipation” characteristic, critical for virtual reality plausibility^36^. The $JNDF_- \le 0.4$ Hz for the “repetitive” attribute at low modulation frequencies (< 5 Hz) corresponds to increased susceptibility to rhythmic “pulsating” stimuli, supporting^19^’s contribution to amplitude-modulation work with stimuli. It also agrees with^37^’s analysis of modulation frequency’s contribution to tactile rhythmic perception. Finally, the “tingling” elicited a $JNDF_- \le 20$ Hz at a 120 Hz reference point, with subjects describing a transition between “tickling” and “smooth,” consistent with^23^’s high-frequency sharpness, and the “up-and-down” $JNDF_- \le 5$ Hz at 30 Hz identifies frequency-modulation perception in terms of a “rising/falling” intensity, important for dynamic tactile cues.

This qualitative distinction is critical for haptic design, particularly for creating “tactons” (tactile icons), as explored by Azadi and Jones^29^. While their work identified that dimensions like frequency, amplitude, and temporal quantities are effective for building a tactile vocabulary, our study provides the quantitative sensitivity data needed to design them effectively. For example, Azadi and Jones identified rhythm as an effective temporal cue; our finding of a $\le 16.7\%$ lower threshold for “repetitive” (a 0.4 Hz $JNDF_-$ at a 2.4 Hz reference) quantifies this sensitivity for the first time. Designers can now use this specific threshold, knowing that a change smaller than 0.4 Hz is likely imperceptible and would fail to create a distinct tacton.

The outcomes of the current research can also be connected to classical psychophysical laws. The methodology, which employed a $log_{10}$ transformation for participant ratings^38,39^, was built on the foundation of Fechner’s law, which shows a relationship between stimulus and sensation. The results for the “weak” attribute support this, showing a consistent symmetric $JNDL_{\pm }$ of $\le$ 2 dB across a 12 dB range (from 14 dB to 26 dB). Since the decibel scale is logarithmic, this constant dB threshold supports a constant Weber fraction for intensity perception. However, the results for frequency-based attributes contradict this for all parameters. For instance, the “repetitive” attribute’s relative threshold increased significantly with the reference modulation frequency, from 16.7$\%$ at 2.4 Hz ($JNDF_-$) to 50% at 5 Hz ($JNDF_{\pm }$). This shows that, while fundamental perceptual magnitudes like intensity may scale according to classical laws,the perception of more complex, temporal attributes like “repetitive” does not. This implies that a single rule (a constant Weber fraction) is insufficient for designing a wide range of haptic experiences. Designers cannot assume that perceptual sensitivity to rhythm or temporal patterns scales in the same way as sensitivity to loudness or brightness.

While the present work brings in a general level of awareness regarding WBV perception, future work will have to expand its scope through examination of additional dimensions of such perceptual descriptors. For example, Seifi & MacLean^40^ demonstrated value in creating tactile feedback libraries that enable perceptual descriptors to map onto physical dimensions, such that specific sensations can be designed for selection by designers. In a similar manner, work in^41^ sees a future for perceptual descriptor rating in defining the shape of haptic feedback systems.

Furthermore, work in^42^ about the ecological approach and tactile transparency in haptic perception postulates that perceptual attributes like “tingling” and “fading” can be utilized to produce contextual, intuitive feedback. In agreement with our experiments, in which perceptual attribute shifts correlated with both JNDF and JNDL thresholds, such work could make multimodal tactile frameworks for haptic systems even more sophisticated, as shown in^43^.

It is also essential to address the limitations of this study, which also serve as opportunities for future research. First, the findings are based on a small sample size (N=11) and second, a disproportionate gender distribution (9 males, 2 females), which constricts the generality of the findings. While this imbalance is a limitation, existing research suggests that gender may not be a significant factor for this type of sensory perception. For this, a study^44^ done for investigating vertical whole-body vibration in recumbent subjects, found no significant differences in the perception thresholds between male and female subjects in their 20s.

In addition to that, this research was conducted in a controlled laboratory environment. Controlled stimulus conditions allowed precise parameters to be set, but these perceptual thresholds may differ when applied to the real world (complex interactions in an automotive system, or immersive virtual environments) where users are processing multiple, additional visual and auditory information at the same time. Future studies should examine how these perceptual attributes are recognized in multimodal contexts.

A further methodological limitation was the use of a subjective rating system (magnitude estimation) rather than a traditional forced-choice or adaptive staircase procedure^45^, which are common methods for JND determination^38^. This methodological choice was used, as the primary goal of the work was not simply to find a detection threshold, but to measure how physical changes transform into perceived, threshold changes in qualitative attributes. Magnitude estimation is a classic method for directly assessing this subjective experience^45^. We acknowledge the primary limitation of this approach: using the variability of a subjective rating as a direct proxy for a JND (a stimulus-level difference) is problematic, as this is “only valid if there is a perfectly linear relationship between stimulus and response”^38^. Subjective rating scales like magnitude estimation are often nonlinear, in part due to the “overly expansive way people use numbers,” which can bias the function^39^. To address this “contaminating output factor”^39^ and to integrate the direct rating method (Stevens’s approach) with discrimination-based scales (Fechner’s approach)^39^, a $log_{10}$ transformation was applied to all participant ratings. As a result, the reported threshold represents a psychophysically-grounded proxy for the JND, defined as the stimulus change required to produce a statistically significant difference in this normalized subjective rating.

In addition, the study did not identify symmetrical JND regions for all of the attributes. For several attributes, such as “weak”, “repetitive”, and “up-and-down”, the very first comparison stimulus tested was already perceived as statistically significantly different from the reference. This indicates that the true perceptual threshold is even smaller than the step we chose. Consequently, the findings for these attributes represent an upper or lower bound of the threshold, not the precise threshold value. This coarse spacing also explains why a symmetrical $JND_+$ region was not identified for attributes like “up-and-down”; the stimuli tested above the reference were likely too far from the reference to identify the upper threshold. Future work is required to map the full perceptual space symmetrically to determine both $JND_-$ and $JND_+$ and explore any potential asymmetries in perceptual discrimination.

## Conclusion

This research presents a series of empirically measured perceptual thresholds, demonstrating how qualitative attributes of whole-body vibration relate to their underlying physical parameters. By identifying and estimating the perceptual discrimination thresholds for “weak”, “up-and-down”, “tingling”, “repetitive”, “even”, and “fading”, this work provides quantitative data that can be incorporated into a framework for linking tactile feedback with human perception. These observations can then be used in diverse applications such as virtual reality or assistive technologies; therefore improving quality of interaction and user satisfaction based designs.

## Data Availability

The datasets used and analyzed during the current study available from the corresponding author on reasonable request.
